# The Impact and Molecular Mechanisms of Exercise in Cancer Therapy

**DOI:** 10.3390/cimb47050374

**Published:** 2025-05-20

**Authors:** Yingjie Sun, Yixiao Ma, Lei Shi, Tong Liu, Yahong Dong, Qiguan Jin

**Affiliations:** College of Physical Education, Yangzhou University, Yangzhou 225009, China; sunyingj@outlook.com (Y.S.); myixiao@163.com (Y.M.); dx120240095@stu.yzu.edu.cn (L.S.); liut9904@163.com (T.L.); dongyahong@139.com (Y.D.)

**Keywords:** exercise, cancer patients, physical activity, exercise prescription

## Abstract

Cancer is a major global health issue, and exercise has become a key supportive treatment. It contributes to reducing cancer risk, enhancing prognosis, and aiding recovery, especially for survivors. However, the exact mechanisms, such as how exercise reduces cancer risk or enhances treatment, are still unclear. Current research often focuses on specific cancer types, ignoring the diverse needs of patients. This limits the development of personalized exercise plans. Additionally, there is insufficient comparison of exercise types—like aerobic, resistance, and high-intensity interval training—regarding their adverse effects and long-term benefits. The best combination of exercises and personalized strategies remains unknown. This review underscores the contribution of physical exercise to cancer prevention and treatment, emphasizing its positive effects on reducing fatigue, improving physical strength, and enhancing mental health. It also explores the molecular mechanisms of regulating tumor immunity and energy metabolism. Additionally, the article covers criteria for selecting exercise types and intensities, and the development of personalized exercise plans. Finally, it provides guidelines for exercise prescriptions and suggests future research directions to improve interventions for cancer patients.

## 1. Introduction

Cancer is a complex multifactorial disease influenced by lifestyle factors (such as unhealthy eating habits, smoking, and physical inactivity), genetic predisposition, environmental exposures, and broader social determinants, including socioeconomic status, culture, policy, and healthcare resources [1,2]. Population aging, lifestyle changes, economic disparities, and other factors are expected to drive a sharp increase in global cancer cases in the coming decades. Research estimates that by 2030, the global new cancer cases will reach 20.3 million [3,4]. Currently, in a broad range of malignancies, prostate cancer (PCa) remains the leading cause of cancer-related mortality in males, accounting for approximately 375,000 global deaths in 2020 [5]. Others include breast cancer (BC, 11.4% of global cancer deaths) [6], colorectal cancer (CRC, 2 million annual new cases) [7], and lung cancer (LC, 18% of global cancer deaths) [6]. The ilk also encompasses hematological malignancies (rising incidence since 1990) [8], head and neck cancer (HNC, 4.8% of global cancer deaths) [9], gynecological cancers (1.3 million diagnoses in 2018) [9,10], and childhood and adolescent cancers (at least 50,000 people under 20 are diagnosed each year in China) [11]. Additionally, less common types, such as pancreatic cancer (PC) [12] and testicular cancer (TC) [13], are also encompassed.

As the country with the highest number of new cancer cases and related deaths [9], China faces enormous health and social pressures [14]. Many cancer survivors endure long-term physical and/or psychological stress due to their illness or treatment [15]. As an example, cancer survivors often face higher recurrence and mortality rates compared to the general population. Furthermore, a quarter of cancer survivors continue to experience fatigue symptoms even years after treatment, making independent living and social reintegration difficult [16]. Finally, some cancer treatments may have side effects. For instance, the risk of developing cardiovascular diseases increases [17,18]. Meanwhile, almost all survivors experience some psychological stress after being diagnosed with cancer [19]. Anxiety, depression, loneliness, fear of recurrence, and body image issues are common challenges faced by patients [20]. Research indicates that the population of cancer patients is expected to continue growing [21]. Therefore, effective strategies are crucial for managing common treatment-related side effects and enhancing both the quality and duration of life after cancer [22].

Exercise, as a safe and effective non-pharmacological intervention supported by theoretical models such as the behavior-change theory [23], the kinetic-chain rehabilitation approach [24], and motor-learning principles [25], enables cancer patients to enhance physical activity capacity while ensuring safety, thereby significantly improving clinical outcomes.

Exercise can offer benefits for cancer patients [26], and there is compelling evidence demonstrating that it reduces anxiety, depression, and fatigue, alleviates sleep disorders, and improves overall quality of life [27,28,29,30]. A systematic review and meta-analysis of 27 randomized clinical trials involving 1929 cancer survivors found that exercise—particularly Tai Chi, yoga, or qigong—can reduce depression and anxiety and improve health-related quality of life (HRQOL) in elderly cancer patients [31]. Moreover, starting exercise before or during cancer treatment can greatly enhance treatment tolerance, functional outcomes, cardiovascular health, and lower the risk of cancer-related mortality and recurrence [32,33,34]. For instance, a meta-analysis of 50,000 CRC and BC patients showed that 150 min of exercise per week could reduce the total risk of death of 24% of BC patients and 28% of CRC patients [35,36]. So, existing evidence suggests potential positive effect of exercise for those side effects associated with the most recent treatments, such as muscle and joint pain caused by chemotherapy, muscle atrophy, cancer cachexia, fatigue, decreased quality of life, and cardiac toxicity [37]. Among them, cardiotoxicity refers to cardiovascular complications caused by cancer treatment drugs. Bodily exercise has been proven to effectively reduce the negative effects of these drugs on the cardiovascular system during and after treatment, and has the potential to prevent cardiac toxicity before clinical symptoms appear [36]. A multifactorial syndrome, cancer-related cachexia is marked by muscle mass loss, which ultimately reduces quality of life and can hinder treatment effectiveness. Exercise serves as an effective adjuvant therapy, alleviating the impact of cachexia on skeletal muscle by regulating mechanisms like protein metabolism, oxidative stress, inflammation, and mitochondrial dysfunction [38].

This review aims to analyze and synthesize the existing literature on the exercise–cancer nexus, exploring its role in cancer patients undergoing surgical and/or pharmacological treatments. Additionally, we delve into the molecular mechanism of exercise inhibiting tumor occurrence and development and provide evidence-based, safe, and personalized exercise prescriptions tailored to distinct cancer populations.

## 2. Exercise-Based Rehabilitation: A Key Pathway to Functional Recovery in Cancer Patients

Cancer rehabilitation encompasses a range of interventions and treatment strategies [39,40]. It is primarily delivered by oncologists and rehabilitation physicians, with a team that typically includes social workers, physical therapists, psychologists, and nurses, along with other specialists to address specific issues faced by cancer patients. Within the limitations of the disease and its treatment, cancer rehabilitation can help patients achieve the highest possible level of physical, psychological, social, and occupational functioning [41].

Studies have shown that exercise-based rehabilitation delivered by trained professionals—such as physiotherapists and exercise physiologists—is safe, feasible, and beneficial in helping cancer survivors return to normal life and adopt a more active lifestyle [42]. A recent study demonstrated that, regardless of tumor type or cancer stage, exercise programs prescribed by physiotherapists and clinical exercise specialists can reduce fatigue and improve aerobic capacity [43]. Major health organizations, like the World Health Organization (WHO), the National Cancer Institute (NCI), the American Cancer Society (ACS) and the Centers for Disease Control and Prevention (CDC), advocate for regular physical activity among cancer survivors after treatment to sustain their overall well-being [14,44,45,46,47].

At present, various exercise models exist to support the rehabilitation of cancer survivors. One approach adopts a group-based model with a fixed exercise schedule; however, due to venue constraints and limited scheduling flexibility, it tends to be less appealing to patients in the acute phase [48]. Another approach involves early assessment and regular follow-up to deliver personalized exercise prescriptions, although this may lead to increased healthcare costs [48].

As is well known, there are various forms of exercise, and different types of exercise stimulate different physiological systems in the body. For example, aerobic exercise primarily promotes lipid metabolism and cardiovascular health [49], while resistance training helps increase muscle size and strength [50]. With social progress and changes in lifestyle, the forms of physical exercise continue to evolve [51]. For instance, the modern societal demand for health and stress reduction has driven the development of exercise forms that emphasize the mind–body connection, such as yoga and tai chi [52]. The following section outlines common exercise modalities along with their respective definitions (Table 1).

## 3. The Role of Exercise Rehabilitation in Cancer Patients

### 3.1. Inflammation

As a non-drug therapy, exercise reduces pro-inflammatory cytokines, thereby lowering inflammation and oxidative stress, while also strengthening the body’s antioxidant system [64].

Animal models investigating exercise-induced anti-inflammatory effects predominantly focus on lymphoma, BC, PCa, and CRC. Research by Zielinski MR et al. [65] demonstrated that daily intense, prolonged exercise delayed the growth of subcutaneously transplanted EL-4 lymphoma tumors in mice, reduced the count of inflammatory cells (neutrophils and macrophages) by approximately 33%. Endurance training can normalize inflammation in tumor-bearing animals [66]. For example, in an anti-inflammatory exercise model for cancer mice, the primary mode of exercise is aerobic activity. Yang, Veras, and LEE et al. [67,68,69] found that aerobic exercise helps to lower pro-inflammatory factor levels (TNF-α, IL-6) in the models of BC and PCa, while significantly increasing anti-inflammatory cytokines (IL-10). Similarly, Emmons et al. [70] demonstrated a decrease in pro-inflammatory cytokines (IL-1β: 0.46 times, IL-1α: 0.89 times, IL-17a: 0.94 times, G-CSF: 0.75 times) following exercise in a carcinogen-induced CRC model.

According to the most recent systematic review and Bayesian network meta-analysis, regular exercise can effectively reduce inflammatory responses in cancer patients, with combined high-intensity aerobic and resistance training proving most efficacious [71]. Human studies on the anti-inflammatory effects of exercise have predominantly focused on PCa and BC patients. Specifically, Kaushik et al. [72] showed reduced inflammation (MCP-1, G-CSF, and Flt-3 ligand) following a 9–12 week supervised yoga exercise program in men with PCa. Moulton [73] and Hooshmand et al. [74] examined the modulation of inflammation markers in post-surgery female BC patients. The results suggest that when following a 12–16-week exercise training program, IL-6 significantly decreased in the exercise group. In addition, Schauer [75] and Hooshmand [74] et al. demonstrated that high-intensity training significantly outperformed low- to moderate-intensity training in reducing serum TNF-α concentrations. Overall, exercise has a positive impact on inflammatory markers, body composition, and health.

### 3.2. Immune Function

Exercise has been shown to modulate inflammation, which in turn can enhance tumor immunity. The important function of the immune system in oncological rehabilitation has been widely discussed [76], but the mechanisms of anti-tumor immunity have yet to be thoroughly explored [77]. Lv et al. [77] demonstrated that the immune system requires uninterrupted immune response to eradicate tumors, and referred to it as the cancer–immune cycle.

Cytotoxic immune cells, like T cells and NK cells, are an important reason why the immune system can resist cancer invasion. T cell subsets include CD8^+^ T cells (CD3^+^CD56^−^CD8^+^) and NKT-like cells (CD3^+^ CD56^+^) [78]. T cells, functioning as cytotoxic effector cells within the adaptive immune system, play a vital role in the host’s defense against solid tumors [79]. Studies have identified that a specific subset of CD4^+^ FoxP3^+^ T cells, known as regulatory T cells (Tregs), which are responsible for promoting dominant immune tolerance to tumors [80]. The dynamic balance between anti-tumor immune responses and immune suppression can be assessed by the intratumoral ratio of CD8^+^ cytotoxic T cells to FoxP3^+^ Treg cells [81].

**Figure 1:**
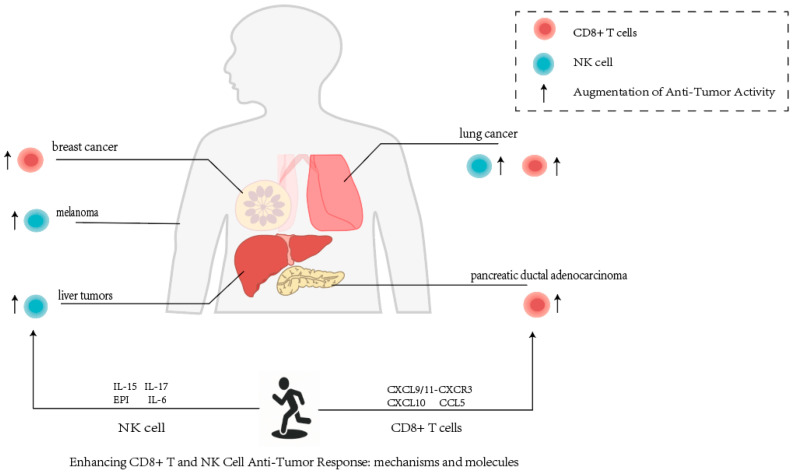
Exercise mediates CD8^+^ T cell and NK cell synergistic antitumor activity in various cancers through multiple molecular mechanisms [87,88,91,93,94].

Sustained high-intensity aerobic training has demonstrated antitumor activity that depends on CD8^+^ T cells in the models of established pancreatic ductal adenocarcinoma (PDA) [82,83], PCa [84,85], liver cancer, and BC [86] (Figure 1). Exercise enhances CD8^+^ T cell recognition via the CXCL9/11-CXCR3 pathway, enhancing T cell receptor (TCR) response to tumor-associated antigens, which contributes to antitumor activity [87]. Miao et al. [88] show that 10 weeks of moderate-intensity exercise increased CXCL10 and CCL5 levels through elevated epinephrine (EPI), which accelerated CD8^+^T cell recruitment contributing to the anti-tumor effect and ultimately inhibiting tumor growth.

NK cell subsets, identified as CD3^−^ CD14^−^ CD16^+^ CD56^dim^ lymphocytes or CD3^−^ CD14^−^ CD16 ^±^ CD56^bright^, have a key role in the innate immune system. These cells are independent of antibody or major histocompatibility complex restriction [78,89,90]. NK cells, an innate immune cell population, are crucial for exercise-mediated tumor modulation. Pedersen et al. [91] showed that physical exercise could increase NK cell anti-tumor activity through mobilization and redistribution in an IL-6 and EPI-dependent manner in various murine tumor models (liver cancer, melanoma (Mel), LC) (Figure 1). Cytokines such as IL-15, IL-6, and EPI may directly inhibit cancer cell proliferation. EPI, the primary hormone in the adrenal medulla, selectively mobilizes cytotoxic cells, promoting immune cell infiltration into tumors [92]. Pedersen et al. [91] suggested that adrenaline can activate the beta-adrenergic receptors on NK cells through exercise, leading to their mobilization into the bloodstream. This process is partially mediated by IL-6 derived from muscles. During skeletal muscle exercise, IL-15 and IL-7 are highly expressed and secreted, playing a role in maintaining T cell homeostasis [93,94]. During exercise, IL-15 acts as a key upstream regulator of CD8^+^ T cells, promoting their survival and the acquisition of a cytotoxic/effector phenotype, which is essential for the anti-tumor effects of exercise [12,95,96].

Studies on the effect of physical activity on tumor immunity in patients primarily focus on PCa, BC, esophageal cancer (EC), and ovarian cancer (OC). Some studies confirm that performing one-time and repeated exercise before radical prostatectomy and during neoadjuvant chemotherapy can increase CD8^+^ T cell concentration in circulation [72]. Lee et al. [97] demonstrated that, after 12 weeks of resistance training, ovarian cancer survivors showed increased sensitivity of CD4^+^ and CD8^+^ T cells, which may facilitate the secretion of myokines and cytokines. Schaueret al. [78] and Parent-Roberge et al. [98] found that, in early-stage PCa patients and metastatic cancer patients treated with chemotherapy, a single high-intensity exercise session, a high-intensity interval exercise session, and a moderate-intensity continuous aerobic exercise session, led to increased NK cell concentration in circulation. Similarly, a six-week program of aerobic and resistance exercise can significantly enhance peripheral natural killer (NK) cell function in patients with stage I–III BC [99]. Additionally, physical activity can induce alterations in immune cell metabolism, though the mechanisms remain to be elucidated [100]. Immune cells regulate the influx of metabolites, metabolic pathways, and cell fate through metabolic reprogramming, promoting the development and progression of tumors [101]. This reprogramming can also alter the composition of extracellular metabolites in the surrounding microenvironment, affecting signal intensity and promoting phenotypic transformation of stromal cells, further promoting tumor growth [102]. Early studies have focused on metabolic pathways regulating tumorigenesis [103]. For instance, in the BC model, seven weeks of endurance training decreased lactate dehydrogenase A expression, increased lactate dehydrogenase B expression, reduced tumor lactate levels, and lowered monocarboxylate transporters expression [104]. These changes reduce lactate production and thereby prevent acidification-induced tumor invasion. Zimmer et al. [105] found that a 12-week supervised resistance training significantly diminished serum kynurenine (KYN) levels and the KYN/TRP ratio. Elevated KYN levels, catalyzed by enzymes like IDO and TDO, suppress immune functions, including T-cell proliferation and cytotoxic activity, thereby promoting tumor progression [106,107]. Exercise-induced reductions in KYN levels mitigate these immunosuppressive effects and improve clinical outcomes.

### 3.3. Energy Metabolism

Tumor cells undergo metabolic reprogramming to meet high biosynthetic demands, relying heavily on aerobic glycolysis, known as the Warburg effect [105,108]. It has been fully recognized as a mark of cancer [109]. This shift enhances lactate production, acidifying the tumor microenvironment and inhibiting T-cell responses [109,110]. Hirschhaeuser et al. [111] designated lactate as a crucial metabolic factor in cancer, contributing to radio-resistance, immune escape, and cell migration [109].

Within animal models, aerobic exercise influences cancer metabolism by reducing glycolysis-related metabolites and increasing HDL levels, potentially improving tumor immune responses [68,112]. For example, Veras et al. [68] found that exercise reduces cholesterol, glucose, and lipid levels, likely by enhancing fatty acid oxidation in muscle cells, which may in turn promote HDL synthesis and improve systemic inflammation and vascular health.

In human studies, cytokines affecting metabolic nodes—Peroxisome proliferator-activated receptor-γ coactivator (PGC-1α), Mitochondrial-derived Peptide MOTS-c (MOTS-c), C-peptide, and the trace element iron (Fe) are commonly detected in patients with PCa, BC, and multiple myeloma (MM). PGC-1α acts as a part in this process, promoting energy metabolism and reducing oxidative stress, which is also supported by randomized controlled trials [70].

In addition, mitochondrial-derived peptides like MOTS-c, which mimic exercise effects, can stimulate glucose utilization, reduce inflammation, and protect against metabolic diseases [113]. A 16-week cardio and strength workout routine increased MOTS-c expression in cancer survivors of non-Hispanic origin, leading to reductions in fat mass, BMI, and improvements in muscle strength and cardiometabolic biomarkers [112]. C-peptide, a byproduct of proinsulin processing, serves as a biomarker for β-cell function. D’Alonzo et al. [114] identified that 12 months of weight training and aerobic exercise reduced insulin and C-peptide levels, suggesting improvements in metabolic function. In MM, exercise also influences Fe homeostasis, with research by Czerwi et al. [60] showing modest changes in serum iron levels after Nordic walking training.

In summary, exercise is recognized as an effective tool for preventing and managing metabolic diseases. It regulates energy metabolism through various mechanisms, such as reducing lipid levels, modulating PGC-1α expression, alleviating adverse effects of ADT, and influencing proinsulin byproducts like C-peptide. Yet, human studies on exercise-induced metabolic adaptations remain limited compared to animal models, primarily due to interindividual variability in metabolic responses (influenced by genetic predisposition, health status, and environmental factors) [115] and technical challenges exist in dynamically monitoring real-time fluctuations—such as lactate levels and resting metabolic rate—during physical activity [116]. Current human investigations predominantly focus on static metabolic parameters such as insulin sensitivity, fasting glucose, and waist circumference [117]. Moreover, to develop precise exercise prescriptions, it is crucial to identify biomarkers and specific signatures that define exercise intensity in animal models—a critical foundation for translational research [118]. Future studies integrating wearable biosensors with digital twin technologies could establish dynamic exercise-metabolism models, enabling personalized exercise intervention design tailored to cancer patients’ metabolic profiles [119]. Future research should explore the signaling molecules involved, such as AMP-activated protein kinase (AMPK), mammalian target of rapamycin (mTOR), and PGC-1α, to improve personalized exercise interventions and enhance clinical outcomes [120].

### 3.4. Hormones and Neurotransmitters

In addition to regulating inflammation and immunity, exercise also influences metabolic growth factors and hormones, which can affect disease progression and outcomes through various mechanisms.

#### 3.4.1. Insulin

Insulin, secreted by beta cells in the pancreatic islets, binds to insulin receptors expressed in all body cells, including tumor cells [121]. Moreover, it binds to the insulin-like growth factor 1 (IGF-1) receptor and is involved in the regulation of cell proliferation and apoptosis [122].

In cancer patients, with a particular emphasis on BC and CRC patients, the effects of exercise on insulin and IGF-1 levels remain inconsistent. For instance, Brown et al. [123] reported that overweight and obese stage I–III CRC survivors showed reductions in fasting insulin levels (27.0 ± 8.3 pmol/L) and insulin resistance (0.63 ± 0.17 pmol/L) following six months of moderate-intensity aerobic exercise. Similarly, Irwin et al. [124] reported that a 6-month moderate-intensity aerobic exercise intervention reduced Insulin-growth factor binding protein 3 (IGFBP-3) and IGF-I levels in BC survivors (3.4% and 4.6%). However, Onerup et al. [125] observed no effect on IGF-1 or IGFBP-3 levels in 217 CRC survivors undergoing a shorter, 30 min daily aerobic exercise regimen before and after surgery. These inconsistencies could be attributed to variations in study design, patient populations, and exercise duration. Future research should prioritize personalized approaches, considering specific patient populations (e.g., those with different BMI or metabolic status), and explore various forms of exercise, like blending aerobic and strength training or incorporating high-intensity interval training, to better understand their effects on insulin and IGF-I levels.

#### 3.4.2. Sex Hormone

Sex steroid hormones play a critical role in the increased cancer risk of postmenopausal women and are modulated by physical activity [126,127,128,129]. Estrogens promote BC cell proliferation and inhibit apoptosis, whereas androgens such as testosterone stimulate cell growth independently of estrogen signaling. Sex hormone-binding globulin (SHBG) can specifically combine with both of them. In BC survivors, elevated levels of estrogen and testosterone correlate with higher cancer recurrence and mortality, whereas elevated SHBG levels are tied to a lower risk of death [126]. Liu et al. [130] used Mendelian Randomization and large-scale Genome-Wide Association Study data to confirm this.

Current research on exercise-mediated modulation of sex hormones has predominantly focused on BC patients. As reported by McTiernan et al. [131], 12 weeks of moderate-intensity aerobic exercise in postmenopausal women reduces serum estrogen, estradiol, and free estradiol levels by 11.9%, 13.7%, and 16.7%, respectively, while increasing SHBG levels by 13.1%. A systematic review of the biological mechanisms suggests that physical activity may reduce the incidence of breast cancer by lowering circulating hormones, thereby exerting a protective effect against cancer [132].

According to research, both aerobic and strength workouts impact sex hormones, with aerobic exercise potentially having a stronger effect [133], which may be attributed to the superior ability of aerobic exercise in reducing body fat percentage, as decreased adiposity is significantly associated with reduced estrogen levels, particularly in postmenopausal women [134]. However, it is worth noting that studies by Brown [135] and McTiernan [136] found that exercise interventions did not significantly alter serum concentrations of sex hormones, although estradiol decreased by 8% and SHBG increased by 2.4%. Variations in baseline hormone levels, age, genetic factors, study durations, and sample sizes may contribute to these inconsistencies.

In summary, the above studies indicate that exercise may slow the growth of cancer cells and minimize the likelihood of recurrence by increasing SHBG and lowering estrogen and androgen levels. Future research could identify the best exercise routine to boost SHBG and reduce free sex hormones. Additionally, personalized exercise prescriptions based on individual hormone profiles may offer a promising direction in cancer prevention and survivorship care.

#### 3.4.3. Irisin

As a muscle-derived factor secreted during exercise, irisin may exert a beneficial impact in treating a wide range of cancers [137]. It may have a role in cancer caused by metabolic disorders by regulating the metabolism and mitochondrial biosynthesis of muscle cells and adipocytes. Irisin may inhibit tumor occurrence and development by regulating lipid metabolism, increasing cell apoptosis, and suppressing inflammation and metastasis. Irisin levels may serve as a key diagnostic biomarker for BC, kidney cancer (KC), and CRC [138].

Animal studies on irisin typically involve continuous and intermittent aerobic exercise. Lee [139], among others, showed that within a BC model, eight weeks of aerobic or intermittent exercise increased irisin levels and significantly reduced tumor growth, number, and volume. Through in vitro research, Alizadeh et al. [140] discovered that in OC cells, irisin inhibited growth and malignancy through the HIF-1α signaling pathway. This pathway is involved in regulating glucose uptake and lactate conversion, modulating the tumor microenvironment [141]. Irisin also has a key role in central nervous system (CNS) tumors. In a glioblastoma multiforme model, irisin reduced tumor volume by 85% and decreased cell invasiveness by 33%. Irisin induced G2/M cell cycle arrest and upregulated p21, inhibiting cell proliferation and suppressing invasion by upregulating TFPI-2 [142].

Studies on the relationship between exercise and irisin have primarily focused on BC and PCa. Somayeh et al. found that 12 weeks of moderate continuous exercise enhanced the circulating insulin levels in BC survivors [143]. However, Kim and colleagues discovered that transient exercise did not lead to a substantial change in serum irisin levels in PCa patients [144]. The conflicting results across studies are likely due to tissue specificity and other factors such as experimental methods, reagents, tumor tissue handling, and exercise protocols [145,146]. To fully leverage irisin in cancer prognosis and therapy, sequential studies are needed to characterize its expression across diverse tumor types and to elucidate its receptors, signaling pathways, and underlying mechanisms.

#### 3.4.4. Dopamine

Dopamine (DA), a neurotransmitter regulated by central and peripheral brain cells, is closely linked to movement and has anti-tumor properties [147]. Recent studies indicate that DA is critical in inhibiting cancer progression [148]. According to their pharmacological properties, DA receptors are divided into two categories: D1-like receptors such as DR1 and DR5, and D2-like receptors including DR2, DR3, and DR4 [149]. Research on dopamine’s effects primarily focuses on animal models of liver cancer. Zhang et al. [147] observed that a nine-week swimming exercise, with eight minutes per day, suppressed the lung metastasis of transplanted liver cancer by boosting DA concentration in serum, the prefrontal cortex, and tumor tissue. In contrast, excessive swimming (sixteen and thirty-two minutes/day for nine-week) had the opposite outcome. Earlier studies, such as one by Varshan et al. [150] demonstrated that in LC cells, exercise-induced endogenous DA exerts anti-proliferative effects by restraining the PI3K/Akt pathway. Zaim et al. [151] demonstrated that the synergistic effect between exercise-induced DA release and certain drugs significantly attenuates VPF/VEGF signaling, reduces angiogenesis, and potentially inhibits BC proliferation. The aforementioned study demonstrates that exercise-induced DA exhibits dual regulatory effects in cancer modulation: moderate exercise intervention elevates DA levels, which inhibit LC metastasis and BC proliferation through suppression of the PI3K/Akt pathway and VPF/VEGF signaling; conversely, excessive physical activity may paradoxically promote tumor progression.

More research is necessary to comprehensively understand the impact of exercise intensity on tumor progression and to ensure that the therapeutic benefits of exercise do not come with the risk of adverse effects from overtraining. Future studies should further explore the interaction between DA and other signaling pathways (e.g., PI3K/Akt pathway) in exercise-induced anti-tumor effects. Meanwhile, more clinical studies are necessary to examine the impact of exercise intensity and DA in cancer control, ensuring that exercise protocols maximize therapeutic effects without promoting negative outcomes.

### 3.5. Apoptosis

Exercise induces apoptosis in cancer cells by activating molecular pathways like caspase-3, a key driver of programmed cell death. This activation takes place via two primary pathways: the extrinsic death receptor pathway (e.g., FAS/FASL interaction [152]) and the intrinsic mitochondrial pathway [153,154]. Moreover, exercise can adjust the pro-apoptotic and anti-apoptotic proteins. Specifically, exercise upregulates Bax, a pro-apoptotic protein, and downregulates Bcl-2, an anti-apoptotic protein, favoring apoptosis over cell survival, particularly in cancer cells.

In animal studies, the effects of exercise on apoptosis have been well-established, with its impact observed in hepatocellular carcinoma (HCC), LC, BC, PCa, CRC, PC, and multiple myeloma (MM). Research has shown that aerobic exercise significantly upregulates caspase-3 and Bax, while it downregulates Bcl-2 in mice with HCC, BC, LC, CRC, and PCa [155,156]. Furthermore, exercise enhances the expression of p53, which induces apoptosis [157]. Elevated p53 levels have been linked to the pro-apoptotic effects of exercise in cancers like LC and Mel [156,158]. Finally, exercise influences ceramide metabolism, particularly increasing levels of C16-ceramide, which stabilizes and enhances the pro-apoptotic function of p53 [159]. Research indicates that exercise may modulate ceramide metabolism differently depending on cancer type and subtype, as demonstrated by differences in ceramide accumulation between Mel models. Lee et al. found that after two weeks of treadmill exercise, CerS6 was upregulated, and Sphk1 was downregulated in B16F10 Mel mice [158]. Additionally, compared to the BP Mel model, exercise increased the C16 ceramide content in B16F10 tumors [158].

Studies on exercise-induced apoptosis in patients primarily focus on cancers such as PC, PCa and CRC. Whole-body electromyostimulation (WB-EMS) is regarded as a strength training method, typically applied to late-stage cancer patients, aiming to offset muscle loss caused by the disease. Schwappacher et al. [160] provided evidence that a 12-week WB-EMS training program substantially upregulated caspase-3/7 expression, thereby facilitating apoptosis in PC cells. Similarly, in patients with CRC and PCa, 12- week of WB-EMS training resulted in the upregulation of BID mRNA, a key protein involved in initiating apoptosis signaling by engaging Bcl-2 [161]. This exercise regimen also led to a significant increase in caspase-7 mRNA expression. Caspases, sequentially activated, are crucial players in apoptosis. Schwappacher et al. [162] further showed that WB-EMS inhibited cancer cell development and concurrently activated apoptotic pathways in vitro, providing compelling evidence that exercise modulates genes related to cancer cell proliferation and viability.

In summary, exercise can effectively induce cell apoptosis through various pathways. These include the activation of caspase, the equilibrium between pro-apoptotic and anti-apoptotic proteins, and ceramide metabolism. Although these molecular pathways collectively demonstrate how exercise effectively promotes cell apoptosis and inhibits tumor growth, changes in cancer subtypes, exercise types, and model systems may lead to different outcomes in specific protein expression and ceramide accumulation. Collectively, exercise may serve as an adjuvant strategy for cancer therapy by promoting apoptosis and suppressing tumor growth.

### 3.6. Tumor Vascularity

The vascular system plays a central role in delivering chemotherapy, immunotherapy and targeted agents [163]. In tumors, an imbalance between pro- and anti-angiogenic signals creates abnormal vessels and impairs perfusion [164]. This reduces oxygen and nutrient delivery and waste removal, thereby promoting tumor progression [165]. Vascular endothelial growth factor (VEGF), the key regulator of angiogenesis [163], is intimately linked to malignant behaviors such as proliferation, survival and migration of cancer cells [166].

Animal studies have shown that exercise can normalize tumor vasculature by modulating VEGF expression. In BC and melanoma (Mel) models, Gomes-Santos [167] and Savage et al. [168] observed that low-to-moderate intensity exercise increased vessel length and density, fostering vascular normalization. In LC [169] and OC models [170], eight weeks of moderate treadmill running combined with resistance training significantly suppressed VEGF expression and delayed tumor growth. Similarly, Patel et al. [171] demonstrated that twenty weeks of voluntary wheel running effectively reduced VEGF levels in prostate cancer-bearing mice.

In patients with oesophageal cancer, Zylstra et al. [172] reported that structured exercise interventions during neoadjuvant chemotherapy markedly lowered circulating VEGF concentrations. These studies suggest that regular exercise remodels the tumor vascular network—reducing pro-angiogenic factor expression and enhancing vessel density—to induce vascular normalization and ultimately delay tumor onset and progression [163].

### 3.7. Cancer Tissue Invasion and Metastasis

Metastasis is a gradual, intricate process where tumor cells depart from the original tumor, carried by the lymphatic or blood vessels, and ultimately form new tumor foci in distant organs or tissues [173]. Metastatic cancer is a systemic disease that typically causes death by directly colonizing and damaging organ function or by altering metabolism through secretion. Metastatic tumors often develop acquired resistance to existing therapies, making them difficult to treat. Even within the same patient, the response to systemic treatment can differ significantly between primary and metastatic diseases. The tumor microenvironment (TME), epithelial–mesenchymal transition (EMT), and angiogenesis all play pivotal roles in tissue invasion and metastasis [174].

#### 3.7.1. Exercise Inhibits Cancer Metastasis: Animal and Human Studies

Aerobic exercise is commonly studied in animal models of metastatic cancer, where voluntary running has been shown to reduce both the weight and number of metastases by 88% and 34%, respectively, in murine PCa [175]. Murphy [176] and Zhang et al. [147]. found that moderate exercise inhibited lung metastases in B16 MM and transplanted liver cancer by enhancing macrophage antitumor cytotoxicity and activating DR2. However, 16 and 32 min prolonged swimming training led to a 75.5% and 95.3% increase in lung metastasis, highlighting that intense exercise can have the opposite effect [147]. After 7 weeks of voluntary wheel running, PC-bearing mice exhibited reduced expression of pro-metastatic genes, including MMP2, HGFR, and IGF1R [175]. In comparison with the control group, exercise increased the expression of CXCR4 level by about twice. This may be due to the differential impacts of exercise on the TME. CXCR4 has a key role in tumor cell migration and homing to metastatic sites, and exercise-induced increases in blood flow, oxygenation, and tissue perfusion may enhance the recruitment of immune cells, indirectly upregulating CXCR4 expression as part of a stress-adaptive response [175]. These data indicate the subtle influence that exercise intensity can exert on the course and spread of tumors.

In cancer patients, especially those with CRC, exercise has been studied for its effects on tumor invasion and metastasis. Circulating tumor cells (CTCs) are tumor cells that have sloughed off the primary tumor and extravasate into and circulate in the blood [177]. The early detection and characterization of CTCs are important to monitor and prevent the development of metastases [178]. Brown et al. [179] found that stage I-III CRC patients undergoing six months of moderate- and high-intensity aerobic exercise (150 and 300 min/week, respectively) exhibited a significant reduction in CTCs. The liver is the main site of metastasis in CRC, and liver involvement is a major cause of mortality in advanced CRC patients. Wan et al. [180] revealed RPS4X as a potential regulatory molecule and found that a decrease in RPS4X expression, achievable through exercise, resulted in a reduction in metastasis and an increase in cell proliferation and migration in CRC. Cell division cycle-associated protein 4 (CDCA4), a protein involved in regulating the cell cycle, has a key role in cancer development [181,182]. In osteosarcoma, aerobic exercise lowered CDCA4 expression compared to anaerobic exercise, thereby hindering cell proliferation and migration [183]. Sheinboim et al. [184] also demonstrated through an epidemiological study that high-intensity exercise significantly reduces the incidence of highly metastatic cancers, with a 73% risk reduction compared to sedentary cohorts.

**Figure 2:**
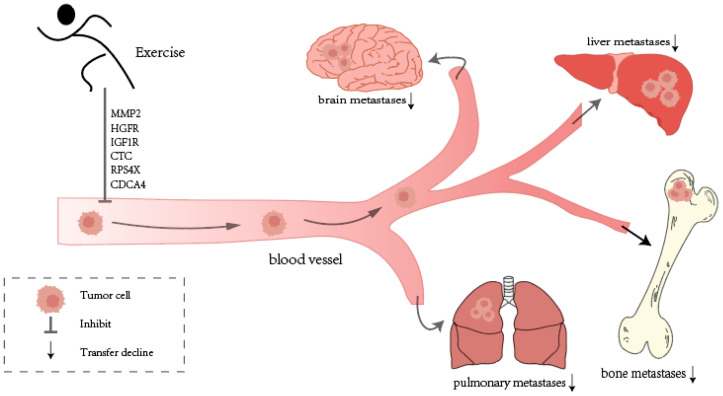
Exercise may suppress cancer metastasis to common organs (e.g., lungs, liver, bones) through the regulation of specific genes and molecules [175,177,180,183].

In summary, aerobic exercise reduces cancer cell growth, migration, and invasion by regulating key molecules like pre-metastatic genes, tumor stemness markers, and RPS4X. It is more effective than anaerobic and high-intensity exercise (Figure 2).

#### 3.7.2. Challenges and Solutions in Metastatic Tumors

As a non-pharmacological strategy, exercise is often combined with conventional treatments like chemotherapy, surgery, and radiation therapy. Emerging therapies, such as targeted therapy, immunotherapy, tumor vaccines, nanotechnology, oncolytic virus therapy, and antibody-drug conjugates, have significantly advanced cancer treatment. However, challenges persist in treating metastatic tumors, including drug resistance, tumor adaptability to new environments, limited animal models [185], and insufficient biomarkers for early detection [186]. Metastasis involves complex mechanisms enabling cancer cells to adapt, evade immunity, and colonize distant organs. Future strategies must focus on developing powerful algorithms to analyze big data, engineering advanced organoid and animal models [186], improving detection thresholds, and expanding biomarkers like CTCs, proteins, and metabolites [185]. Finally, artificial intelligence holds promise for transforming biomarker discovery, drug development, and clinical trial design [186].

### 3.8. Body Function and Composition (Human)

Exercise not only affects tumor biology and endocrine system function but also plays an indispensable role in patient health management. The study of exercise in cancer sufferers primarily focuses on improving body function and composition in individuals with TC, HNC, and BC. Supervised exercise training can significantly enhance strength, endurance, flexibility, and overall function [187,188]. Similarly, Samuel et al. [189] discovered that an 11-week aerobic and strength exercise regimen significantly boosted physical function among HNC survivors, as assessed by the 6 min walk test (6MWT). The exercise group demonstrated an improvement of 37 m, whereas the control group saw a decrease of 73 m. Although there have been many research results on postoperative exercise rehabilitation in enhancing physical function, there is a relative scarcity of studies evaluating the outcomes of preoperative exercise. However, existing research suggests that preoperative exercise can also bring positive effects. Triguero-Cánovas et al. [190] found that incorporating daily aerobic exercises and thrice-weekly muscle endurance sessions during the preoperative period diminished postoperative complications (17.4% vs. 33.3%), increased vital capacity, and shortened hospital stays, along with a significant improvement in the 6MWT. The decline in bodily functions due to inactivity is strongly associated with higher rates of postoperative complications, prolonged recovery, and reduced QOL.

For BC patients, shoulder morbidity following surgery, including pain, functional limitations, and decreased range of motion (ROM), is a common side effect [191]. Klein et al. [192] demonstrated that physical therapy can help reduce fatigue, pain, and promote physical functioning and ROM (146 ± 21°) during recovery from major surgeries. Nevertheless, research indicates that minor surgery does not significantly impact ROM, possibly due to less trauma and retention of near-normal physical function. This highlights the importance of tailoring rehabilitation programs according to the nature and extent of surgical stress, with more intensive interventions for major procedures. In TC, exercise reduces the risk of pneumonia and thromboembolic events, which become more likely as patients undergo chemotherapy. Van et al. [193] found that conducting 12 weeks of aerobic and strength exercise program during chemotherapy significantly increasing pulmonary function, von Willebrand factor and factor VIII, reducing pulmonary and vascular endothelial toxicity. With the progress of research, in addition to common forms of exercise, new exercise techniques have emerged, such as the 12-week novel strength training intervention proposed by Lavigne et al. [194]. Combining eccentric overload and neuromuscular electrical stimulation (NMES) in knee extensors, the research indicates that the cross-sectional area of muscles increased by 18 ± 22%, and the maximum isometric voluntary contraction increased by 19 ± 23%. This study is the first to use eccentric overload and NMES to enhance training stimulation for HNC survivors, all while minimizing energy expenditure. Furthermore, virtual reality (VR) is an effective tool in enhancing patients’ physical function and overall QOL [195]. Basha et al. [196] found that both resistance exercise and VR groups achieved significant improvements in physical functioning, including grip strength and the shoulder’s range of motion in the coronal, sagittal, and transverse planes. However, VR training showed superior outcomes in terms of general health, pain, and vitality. Future studies should investigate the effects of VR-integrated resistance training on physical function (e.g., shoulder mobility and pain) in BC patients, and could compare VR’s therapeutic effects with aerobic/resistance training in other cancer populations (e.g., pediatric cancer patients) to inform clinical practice. It is essential that the type, intensity, frequency, and amount of exercise should be adjusted according to each patient’s physical foundation, health condition, cancer type and stage, and monitoring methods.

### 3.9. Cancer-Related Fatigue (Human)

Cancer-related fatigue (CRF) is a complicated, multidimensional clinical manifestation, influenced by physiological, psychological, and disease-specific factors. Its occurrence is closely linked to the abnormal expression of inflammatory factors released by tumors, such as IL-1, IL-6, and TNF-α [197]. In addition to physical pain, patients also face emotional and economic pressures, which exacerbate fatigue. The evaluation of CRF often relies on scales like the Piper Fatigue Scale [198], the Multidimensional Fatigue Symptom Inventory (MFSI-SF) [199], and the Fatigue Severity Scale (FSS) [200].

Research shows that exercise is an effective strategy to combat CRF, particularly in patients with HNC, PCa, BC, CRC, and nasopharyngeal cancer. A recent meta-analysis by Liang et al. [201] has demonstrated that regular exercise adhering to the American College of Sports Medicine (ACSM) guidelines—particularly when adherence is high—provides an effective means to mitigate cancer-related fatigue.

Progressive muscle relaxation (PMR) has emerged as a beneficial therapy for mental fatigue. Emotional tension often leads to muscle tightness, and muscle relaxation can alleviate anxiety by lowering respiratory rate, heart rate, and diminishing sympathetic nervous system activity. This has been confirmed by randomized controlled trials [61] and meta-analyses [202]. While PMR can improve cancer-related mental fatigue, studies have proved that resistance training is more effective than relaxation in alleviating both physical and mental fatigue [203]. Resistance training (RT), particularly when combined with high-intensity interval training (RT-HIIT), had greater efficacy in relieving physical fatigue. It activates type II muscle fibers and induces anti-inflammatory effects. For example, 16 weeks of RT-HIIT reduced IL-6 and soluble CD8a levels in chemotherapy-treated breast cancer patients, improving systemic and physical fatigue by 32.0% and 31.2%, respectively. In contrast, aerobic exercise has a smaller effect on inflammatory markers, likely because it primarily activates type I muscle fibers [204].

Research by Siebert [199] and Demmelmaier et al. [205] also supports the role of high-intensity aerobic and resistance training in improving physical fatigue in PCa, BC, and CRC patients. Additionally, Traditional Chinese Exercise (TCE) like Baduanjin, Qigong, and Tai Chi, which regulate qi and blood (a TCE concept of balancing vital energy and circulation), improve sleep and enhance physical fitness, have been studied for their impact on CRF. Studies by Lu [206] and Cheng [207] show that after 24 weeks, Baduanjin significantly reduced the incidence of severe CRF in CRC patients (from 59.1% to 23.2%). Tai Chi combined with resistance training also demonstrated significant improvements in fatigue after 12 weeks. These findings highlight resistance training’s superior role in combating physical fatigue.

Recent research has broadened the scope of interventions: Dongen et al. [208] observed that partner involvement may promote the alleviation of fatigue, indicating that future work should investigate couple-based exercise intervention models. Concurrently, existing evidence offers a theoretical foundation for intervention methods: resistance training—by activating type II muscle fibers and exerting anti-inflammatory effects—outperforms aerobic exercise in relieving physical fatigue, whereas relaxation therapies such as PMR specifically target mental fatigue by modulating the autonomic nervous system. By combining social support (for example, partner participation) with exercise interventions (such as resistance training) and mind–body therapies (such as Tai Chi), it may be possible to deliver a more comprehensive solution for the multidimensional management of cancer-related fatigue.

### 3.10. Quality of Life (Human)

In terms of patient health, exercise not only has an impact on their bodily functions but also plays a part in enhancing their mentality and QoL. Cancer treatments, including surgery, radiotherapy, and chemotherapy, often lead to severe side effects that adversely affect patients’ QoL, impacting both physical and mental functioning. Even after treatment, many survivors continue to face ongoing challenges. QoL is typically assessed using tools like the EORTC QLQ-C30 [209], which evaluates physical, mental, and social well-being, along with disease-specific questionnaires such as the QLQ-BR23 [210] for breast cancer and FACT-P [211] for prostate cancer.

Aerobic training, strength exercise, high-intensity interval training (HIIT), and mind–body practices like yoga, have shown promise in alleviating cancer-related symptoms, improving physical function, and promoting recovery. The latest meta-analyses indicate that, compared with routine care or inactivity, exercise can significantly enhance mental health, physical function and social interaction, whilst also improving overall quality of life [212]. Sibarani et al. [213] demonstrated that an eight-week program of 15–20 min walks can effectively enhance quality of life in ≥IVa LC patients. Antunes et al. [187] confirmed that a supervised program combining aerobic and strength exercises improved various aspects of QoL in BC patients (physical, psychological, and social functioning). Similarly, Kaushik et al. [72] found that six weeks of yoga significantly improved functional, physical, and social well-being in PCa patients, while laughter yoga also significantly enhanced emotional and role functioning in chemotherapy patients [214,215]. Qigong and Tai Chi, as adjunct therapies to standard care, have also demonstrated beneficial effects on QoL, physical scores (including physical functioning, fatigue and sleep quality) and psychological scores (such as mental health and anxiety) in cancer patients [216].

Despite these positive findings, the most effective exercise intensity for improving HRQoL has yet to be determined. Ax and his colleagues [217] revealed that both high-intensity (HI) and low-to-moderate intensity (LMI) exercises had beneficial effects on HRQoL in patients with CRC, PCa, and BC. LMI participants reported better emotional functioning at 3 months and better global health status at 18 months, while HI participants showed lower respiratory distress at 6 months. The findings indicate that exercise, irrespective of intensity, brings about a positive change in HRQoL within one year of the intervention. HIIT has been shown to be a time-saving substitute for moderate-intensity continuous training (MICT), offering similar metabolic benefits with less time commitment, which could improve adherence. Moreover, HIIT is often more enjoyable than MICT [218], potentially contributing to better long-term adherence [219].

However, not all studies have shown consistent benefits. Morielli et al. [220] found that HIIT during neoadjuvant chemoradiation for rectal cancer negatively impacted role and emotional functioning, underscoring the importance of timing and cancer type in exercise interventions. Similarly, a randomized trial showed that a six-month aerobic training did not significantly improve QoL in PCa patients [221], potentially due to the exercise regimen being unsuitable for this specific group. Future research could explore incorporating resistance training to assess its impact on cancer prognosis.

Nowadays, with the increase in risks of heredity, virus infection, and bad living habits, although the incidence rate of cancer among young people is relatively low, they are still likely to suffer from cancer. Therefore, investigating whether exercise can benefit the QoL of young cancer patients is crucial. Munsie et al. [222] observed that ten weeks of moderate-intensity aerobic, strength, and flexibility training can significant improve the QQL of teenagers and young cancer survivors.

In conclusion, exercise—whether aerobic, resistance-based, or mind–body practices—has shown positive effects on QoL by reducing fatigue, anxiety, and depression in cancer patients. Upcoming studies should include a broader range of cancer patients, particularly those with less common types, and explore the integration of exercise with other QoL interventions to identify the most effective approach for enhancing patients’ quality of life (Table 2).

## 4. Recommended Exercise Prescription for Cancer Patients

Regular participation in moderate-to-vigorous physical activity is recognized as an effective strategy for cancer prevention [223]. It can alleviate treatment-related side effects, improve physiological function, and extend survival [224]. The mechanisms underlying these benefits involve several biological processes: for instance, aerobic exercise increases β-endorphin levels, helping to alleviate stress and pain caused by various conditions [225,226]. Resistance training preserves skeletal muscle mass by optimizing motor unit synchronization and recruitment efficiency, while flexibility training improves joint control and soft tissue adaptability, thereby enhancing functional capacity [227,228].

The American Cancer Society (ACS) and the American College of Sports Medicine (ACSM) jointly recommend that cancer survivors engage in at least 150 min of moderate-intensity or 75 min of vigorous-intensity aerobic exercise per week, supplemented with bi-weekly resistance training and daily flexibility exercises [229]. However, the optimal type, intensity, frequency, and duration of exercise for specific cancer patients remains unclear. While it is unlikely that a tailored exercise prescription will be developed for individual cancer diagnoses, general exercise guidelines are likely applicable to most cancer patients. These guidelines typically follow the FITT (Frequency, Intensity, Time, and Type) principle. Considering the diversity of cancer types, these exercise guidelines aim to ensure safety, feasibility, effectiveness, and individual suitability for each patient (Table 3).

While multi-pattern, moderate to high-intensity exercise is generally suitable for most cancer patients, there is no universal prescription or total weekly amount of exercise. Exercise prescriptions for cancer patients must be targeted and individualized, taking into account patient- and cancer-specific factors [37].

At present, exercise mainly targets the most prevalent adverse effects induced by cancer and treatment, including fatigue, anxiety, depression, lymphedema, decreased QoL, and deterioration of bodily function [240]. However, further research is necessary to assess the role of exercise in promoting other outcomes. Developing specific physical activity (PA) guidelines tailored to different cancer sites, distinct phases of the cancer, and diverse outcomes—such as return to work—is essential for optimizing exercise interventions and personalizing PA programs for cancer survivors [240].

## 5. The Model of Rehabilitation Training for Cancer Patients

The available evidence states that there are various forms of exercise rehabilitation for cancer, which can generally be divided into the following categories: 1. Supervised the exercise rehabilitation plan. 2. Family sports rehabilitation plan. 3. Development personalized plans based on different types of cancer. Supervised training generally yields higher compliance and better outcomes [117,241,242,243]. However, home-based programs are often chosen due to factors like the COVID-19 pandemic, patient preferences, or study designs [244,245,246]. Notably, home programs may lack sufficient intensity and professional supervision, which has been shown to impact effectiveness. For example, a study indicated that family exercise, when used as an adjunct to chemotherapy, did not increase relative dose intensity, likely due to low exercise intensity and lack of professional oversight [247].

Personalized rehabilitation plans are crucial as cancer types vary in anatomy, pathology, progression, and treatment side effects. For instance, BC treatment often causes lymphedema, particularly after surgery and radiotherapy [248], requiring specific exercise recommendations such as wearing compression garments and adhering to intensity guidelines (50–75% VO2peak or 60–80% HRmax for 30–45 min) [249]. PCa treatments, including radical prostatectomy, can lead to erectile dysfunction, libido loss, and urinary incontinence [250,251,252]. Pelvic floor training has been demonstrated to significantly reduce incontinence duration and improve QOL, with early intervention being more effective [253,254,255]. In addition, physical exercise can counteract the metabolic side effects of treatments like androgen deprivation therapy (ADT), improving metabolic syndrome components like fasting blood sugar and abdominal circumference [256,257]. It is worth noting that supervised training is considered more effective than family plans, with supporting evidence from a systematic review of 20 randomized trials and 2188 male participants [258]. CRC survivors often experience a reduction in physical ability, including flexibility, balance, and abdominal muscle strength [259]. This decline not only affects functionality but may also lead to chronic low back pain due to reduced abdominal muscle width [260]. The CO-CUIDATE exercise program has been shown to significantly enhance the physical health, waist circumference, and musculoskeletal conditions related to the lumbopelvic area of CRC patients [117,243].

Currently, exercise monitoring technologies have evolved into multimodal intelligent systems. For instance, Gao et al. [261] have integrated wearable devices, applications, and big data analytics to propose a new intervention approach: mobile health (m-health). Users can utilize smartphone applications to track metrics such as step counts, activity duration, and energy expenditure, while receiving real-time feedback, customized reminders, and goal setting. These devices also allow individuals to share personal data on social networks, thereby fostering self-motivation and peer support.

In summary, cancer rehabilitation strategies can be broadly categorized into three types. Structured supervised training generally improves patient adherence and optimizes clinical outcomes, whereas home-based exercise programs are often chosen due to environmental factors (e.g., transportation barriers) or the patient’s own reasons (e.g., social anxiety disorder) [262]. Personalized plans are critical because cancer types differ in anatomy, pathology, progression, and treatment side effects. While evidence underscores the superiority of supervised training, future research should focus on optimizing home-based exercise programs through AI algorithms (which generate dynamic exercise prescriptions based on individual data) or wearable technologies (such as smartwatches and smart textiles), exploring the benefits of prehabilitation before treatment, and assessing the long-term impact of exercise on survivorship and recurrence rates. Furthermore, the feasibility of implementing intelligent fitness facilities—such as intelligent walking paths and smart fitness stations—could be explored. These systems not only enable the collection and analysis of data across various scenarios but also enhance workout efficacy through integrated recreational and leisure experiences. Finally, understanding how exercise interventions address the specific needs of cancer patients is critical for advancing cancer care and improving patient prognosis.

## 6. Discussion

Cancer is a chronic disease requiring long-term management, with high mortality and morbidity rates. While advancements in treatment have improved survival rates, a lot of cancer survivors face long-term physical, psychological, and metabolic challenges. Exercise, as a non-pharmacological intervention, helps to reduce treatment side effects, enhance immune function, regulate metabolism, and enhance quality of life. Animal studies have revealed that exercise regulates cancer through mechanisms such as suppressing inflammation (e.g., TNF-α, IL-6), enhancing anti-tumor immunity (CD8^+^ T cells, NK cells), promoting apoptosis (Bax/caspase-3), and inhibiting metastasis-related genes (MMP2, IGF1R), with some mechanisms preliminarily validated in humans. For instance, Luo et al. [263] found that voluntary exercise suppressed extracellular matrix remodeling in lung cancer via muscle-derived miR-29a-3p, thereby enhancing immune infiltration and treatment response; this miRNA was also significantly upregulated in exercising individuals. In a pancreatic cancer (PC) model, aerobic exercise activated the IL-15/IL-15Rα axis to drive CD8^+^ T cell infiltration, with similar immune responses observed in clinical samples [12]. Nevertheless, findings from animal studies may not be directly applicable to clinical practice. For example, Mark et al. [264] reported that exercise increased plasma decorin levels—a key factor in suppressing tumor proliferation and metastasis—and reduced breast cancer burden in mice; however, tumor volume changes in patients cannot be directly assessed due to ethical constraints, creating a gap in mechanistic evidence. Furthermore, tumor biology differs between species. Most rodent studies use rapidly growing allograft, xenograft, or genetically engineered models that lack the intratumoral heterogeneity and complex stroma typical of human cancer [265]. There are also notable size-related physiological differences—such as heart rate, with mice averaging 600 bpm compared to 80 bpm in humans—which influence drug metabolism and clearance [266]. Moreover, preclinical studies often use young mice (equivalent to adolescent humans), overlooking the possibility that different tumors may develop at different life stages, further complicating clinical translation [267].

Future preclinical models should focus on simulating the real conditions of cancer patients, use age-appropriate mouse models according to specific cancer types, delve into gene-editing technologies to better mimic the impact of tumor activity on bodily systems, provide detailed reports on reasons for mouse euthanasia, and employ existing technologies to monitor for any abnormal mouse behavior. Moreover, to ensure broad applicability of the results, it is essential to conduct large-scale, multi-center, randomized controlled trials across diverse patient populations. Ongoing studies are necessary to assess the long-term benefits of exercise for cancer survivors, especially in reducing cancer recurrence rates, improving overall survival rates, and preventing cancer-related complications. Lastly, more in-depth research must be conducted to explain the molecular mechanisms by which exercise regulates the TME, immune function, and metabolic homeostasis.

In clinical practice, exercise physiologists and physical therapists must have the expertise to develop individualized exercise plans based on cancer type, treatment stage, and patient needs. Collaboration with other healthcare professionals is crucial to integrate exercise with other treatments, such as medication, and nutritional support, to enhance overall patient care.

With advances in technology, artificial intelligence (AI) can optimize rehabilitation plans by analyzing patient physiological data and treatment feedback. Wearable devices can monitor activity levels and physical recovery in real-time, while symptom prediction models can identify potential risks and provide targeted interventions. Upcoming research should concentrate on exploring the potential of AI, wearable devices, and VR to develop real-time, adaptive exercise monitoring and guidance systems for cancer patients.

Recent research indicates that physical activity of any duration confers positive effects on overall health [268]. Exercise snacks, as an emerging intervention strategy, involve performing multiple brief bouts of exercise (each lasting ≤ 1 min) at intervals of 1–4 h throughout the day [268]. These may include aerobic movements such as one minute of stair climbing, strength activities like hanging out laundry, balance exercises, or combinations thereof (for example, carrying heavy shopping bags), and can even be woven into short intermittent daily tasks (such as tiptoeing to clean windows) [269]. For individuals unable to sustain longer exercise sessions, this approach offers unique appeal and feasibility, and studies have confirmed its safety and efficacy in older adults [269]. In addition, Kaarin et al. [270] proposed another new type of exercise regimen called the “weekend warrior”, which, similar to traditional regimens, is beneficial to brain health. The “weekend warrior” specifically refers to concentrated physical activity on weekends that meets or exceeds the weekly recommended levels of physical activity. Future research could explore the feasibility and effectiveness of implementing these exercise regimens in cancer patients.

Apart from the aforementioned recommendations, public health initiatives and policy implementation should prioritize integrating exercise counseling into standard oncology care and incorporating exercise-based rehabilitation programs into healthcare systems. Educational campaigns should also be launched to raise awareness of the advantages of physical activity in cancer prevention and survivorship. By addressing these key issues, exercise can serve as a fundamental component of comprehensive cancer care, helping patients recover and reducing the global burden of cancer.

## Figures and Tables

**Table 1:** Common exercise modalities and their definitions.

Exercise Types	Definition	References (Refs)
Aerobic exercise	Aerobic exercise refers to a type of physical activity involving continuous, rhythmic movements that elevate the heart rate and engage large muscle groups. Examples include cycling, jogging/running, swimming, and walking.	[53]
Resistance training	Resistance exercise refers to a form of physical activity where muscles contract to counteract an external force, with the goal of improving muscle strength, endurance, or motor performance. Common examples include weightlifting, push-ups, and pull-ups.	[54]
High-intensity interval training (HIIT)	HIIT refers to a type of exercise, which consists of brief bouts of vigorous (70–90% of maximal heart rate) to high-intensity (≥90% of maximal heart rate) exercise lasting from 60 s to 8 min, alternating with recovery periods ranging from 1 to 5 min. These recovery intervals can involve active recovery (40–70% of maximal heart rate) or passive recovery (complete cessation of movement).	[55]
Yoga	Yoga refers to a type of physical activity in which physical postures, breath control, meditation, and ethical principles are integrated to promote health and relaxation.	[56,57]
Traditional Chinese Exercise (TCE)	TCE refers to therapeutic physical activities in traditional Chinese medicine, emphasizing the mind–body connection, slow movements combined with deep breathing, and muscle stretching with mental concentration. Examples include Qigong, Tai Chi, and Baduanjin.	[58,59]
Nordic walking training	Nordic walking, also known as walking with poles, refers to a type of physical activity that is a safe option recommended for both older individuals and patients with hematologic malignancies.	[60]
Progressive muscle relaxation (PMR)	PMR refers to a type of mind–body relaxation technique in which specific muscle groups are contracted and relaxed to reduce muscle tension and alleviate anxiety.	[61]
Pelvic floor muscle training (PFMT)	PFME refers to a type of exercise aimed at improving the strength, endurance, and power of the pelvic floor muscles. It can effectively enhance pelvic floor function and prevent urinary and fecal incontinence, and is commonly used in patients with CRC and other pelvic malignancies.	[62]
Inspiratory muscle training (IMT)	IMT was defined as any intervention with the goal of training the inspiratory muscles.	[63]

**Table 2:** Classify and summarize the research findings in Section 3 by species, type of exercise, specific training program, type of cancer, and research outcomes.

	Species	Exercise Mode	Specific Exercise Plan	Cancer	Finding	Refs
Inflammation	Mice	Endurance training	Exercised 5 days a week for 1 h at 5° incline, velocity gradually increasing to 20 m/min over 2 weeks, 5 weeks.	LC	IL-1β ↓	[66]
		Aerobic exercise	Ran for 1 h per day at any time during the period.	PCa	IFN-γ ↓ TGF-β ↓ TNF-α ↓ IL-4 ↑ IL-10 ↑	[67]
		Aerobic exercise	Ran for 30 min, at a speed of 18 m/min for 5 days per 12 weeks	BC	TNF-α ↓ IL-6 ↓ CRP ↓	[69]
		Voluntary wheel running	10% incline, speed from 6 m/min to 33 m/min, for 4 weeks.	BC	TNF-α ↓	[70]
	Rat	Aerobic exercise	For 8 weeks, 5 sessions per week, at 59% of maximum capacity.	PCa	TNF-α ↓ NF-κB ↓	[68]
	Human	Yoga	60 min of yoga twice weekly for 6 weeks.	PCa	inflammation ↓	[72]
		Walking test	6–8-week; 45–60 min	Child hood cancer	SII ↓	[73]
		Community-based and Acute exercise	40–60 min, 10–30 min of aerobic exercise, and 30 min of resistance training.	BC	BCS ↑ IL-8 ↑	[75]
Immune Function	Mice	/	/	BC	CD8 T cells ↑ CXCR3 ↑	[87]
	Human	Yoga	60 min, twice weekly for 6 weeks	PCa	CD4^+^ and CD8^+^ T-cells ↑	[72]
		Resistance exercises	4 days a week for 12 weeks	OC	CD4^+^ and CD8^+^ T-cells ↑	[97]
		Acute exercise	A watt-max test and four high-intensity intervals	PCa	NK ↑ NKT-like ↑ CD8 T cell ↑ Granzyme-B ↑	[78]
		HIIT	4–6 cycles	PCa	NK-cell ↑	[92]
		Resistance exercise	12-week; twice a week	BC	KYN levels ↓ KYN/TRP ratio ↓	[105]
		Aerobic and resistance exercises	six-week	BC	NK-cell ↑	[99]
Energy Metabolism	Human	Nordic Walking Trainings	6 weeks, 60 min/day (5 min warm-up, 45 min main part, 10 min cool down).	MM	Fe ↓	[60]
Dopamine	Mice		8 min/day, 9 weeks	Liver cancer	DR2 ↑	[147]
Sex hormone	Human	Aerobic exercise	Thrice-weekly; 8-week	BC	Free estradiol ↓ Estrone sulfate ↓ Total testosterone ↓ Androstenedione ↓ Dehydroepiandrosterone ↓	[136]
Irisin	Mice	Wheel running	3 weeks, 6.1 km/mouse/day	Glioblastoma	Irisin ↑	[142]
Apoptosis	Mice	Aerobic exercise	At 12 m/min for 45 min for 5 consecutive days; with 2 days of rest per week for 2 weeks	Mel	MDM2 ↓ CerS6 ↑ Sphk1 ↓ C16-ceramide ↑ The total ceramide concentration ↓ 30.4%	[158]
		Aerobic exercise	1 h per day for 21 days	PCa	Bax ↑ Caspase-9 ↑ Caspase-3 ↑ Bcl-2 ↓	[67]
	Human	Resistance exercise	20 min/session, 2 sessions per week, for 12 weeks	PCa; CRC	HT29 cells ↓ 10.6% LNCaP cells ↓ 7.7% PC3 cells ↓ 7.4%	[162]
Cancer Tissue Invasion and Metastasis	Mice	Voluntary wheel running	Voluntarily use wheels 24 h a day, 8 weeks	PCa	CXCR4 ↑ twofold IGFR-1 ↓ MMP2 ↓	[175]
		Swimming	8 min/day, 9 week	Liver cancer	Weight ↓ 19.4% Lung metastases ratio ↓ 34.3% DA ↑ 68.3%	[147]
Body Function and Composition (Human)	Human	Family exercise	Flexibility exercises, wall push-ups, and upper body resistance exercises three times a week for 30 weeks.	BC	The 2 min step test ↑ The back scratch test ↑ The timed bicep curls test ↑	[188]
		Aerobic and resistance exercises	11-week, with 5 sessions per week at an intensity of 3–5/10 on the RPE scale.	HNC	6MWT ↑ 37 m. Physical Component Score ↑ 10.5% Fatigue values ↑ 33.78%	[189]
		Therapeutic exercises and Home exercise	Three times a day, five repetitions of each exercise, for six months.	BC	Pain values (NPRS) ↓ The flexion ROM ↑ The extension ROM ↑	[192]
		VR training and Resistance exercises (RE) training	8 weeks, 5 times a week, with 50% to 60% of the maximum number of repetitions of game based training.	BC	VR Training Group: VAS scores ↓ DASH scores ↓ Shoulder flexion ↑, Abduction ↑ Eternal rotation ↑ RE Training Group: Shoulder flexion ↑ Abduction ↑ External rotation ↑ Handgrip strength ↑	[196]
		Home-based prehabilitation Aerobic exercises and Endurance exercises	Daily aerobic exercises and 3 weekly sessions of each lasting 30 min, 6 weeks	CRC	Postoperative complications ↓ 6MWT ↑ + 78.9 m Ergospirometry ↑ 1.5METs	[190]
		Eccentric overloaded strength training and Neuromuscular electrical stimulation	2 sets of 8 repetitions of unilateral squats, 12 weeks.	HNC	MIVC ↑ Cross-sectional area ↑ Cycling exercise time ↑	[194]
Cancer-Related Fatigue (Human)	Human	RT-HIIT AT-HIIT	60 min exercise sessions twice weekly for 16 weeks.	BC	IL-6 ↑ CD8a ↑	[204]
		/	60 min each session; twice a week for 12 weeks.	NC	General fatigue ↓ Physical fatigue ↓ Emotional fatigue ↓ Mental fatigue ↓ QOL ↑ Physical function ↑ Social function ↑ Role function ↑	[203]
		Endurance and Strength Training	3 times a week, lasting 30 min, for a period of 4 weeks	BC, CRC PCa, Lymphoma	General fatigue ↓ physical fatigue ↓	[199]
		Resistance training and home-based endurance training	6-month, resistance training twice a week, 150 min of moderate to low-intensity walking or cycling per week.	BC, CRC PCa	Physical fatigue ↓ Cardiorespiratory fitness ↑ Leg strength ↑	[205]
		Baduanjin Qigong	At least 5 times a week, for 20–40 min each time, 24 weeks	I-III CRC	The KPS scores ↑ The PSQI scores ↓	[206]
		Tai Chi and Resistance Training	12-week (3 days per week), 40 min per day	LC, BC, Gastric cancer	GAD-7 scores ↑ PHQ-9 scores ↑ the PSQI scores ↓ the QLQ-CCC ↑	[207]
		Aerobic and resistance exercises	Aerobic: 150 min/week Resistance: Two times a week	Cancer	CRF ↓	[201]
QOL (Human)	Human	Aerobic and resistance training	Three times a week	BC	QoL ↑ QLQ-C30 ↑	[187]
		Yoga	60 min each time, twice weekly for 6 weeks	PC	QoL ↑ FACT-physical well-being ↑ FACT-Social wellbeing ↑	[72]
		Laughter yoga	Four weeks, four times a week, 20–30 min each time	Cancer	Emotional function ↑ Role function ↑ QoL ↑	[215]
		Aerobic, Strength, and Flexibility Exercise	Twice a week, 60 min each time, 10 weeks	Cancer	the ADS total score ↓ Anxiety score ↓ QOL ↑	[222]
		HIIT and MICT	Three times a week, 12 weeks	BC	The physical well-being ↑ Social well-being ↑ Emotional well-being scores ↑ Functional well-being subscale scores ↑ FACT-G scores ↑ Functional well-being subscale scores ↑	[218]
		Walks	15–20 min, 8 weeks	LC	QOL ↑	[213]
		Qigong and Tai Chi	/	Cancer	QoL ↑ physical functioning ↑ sleep quality ↑ Mental health ↑ Anxiety ↓	[216]

**Table 3:** Exercise prescriptions for different cancer patients.

Exercise Types	Suitable Population	Applicable Phase	Exercise Intensity	Frequency/Duration	Period	Refs
Aerobic exercises	Adult cancer patients	Postoperative/Late stage patients	Low	5–10 min Accumulate at least 20 min	/	[37]
Aerobic and resistance exercises	Adult cancer patients	/	Moderate	Aerobic: 150 min/week Resistance: Two times a week	As defined by the patient	[230]
Aerobic and resistance exercises	Adult cancer patients	/	High	Aerobic: >75 min/week Resistance: two times a week	As defined by the patient	[230]
Aerobic exercises	CRC patients	After post-primary cancer treatment (surgery, chemotherapy, radiotherapy)	Moderate	>150 min/week; Three to seven times a week	≥12 weeks	[231,232]
Aerobic and resistance exercises	BC Patients	Within five years of treatment/after treatment completion	Moderate	50 min each time; Three times a week	12~26 weeks	[233,234]
Aerobic, resistance, and impact exercises	PCa patients	patients with locally advanced and metastatic PCa	Moderate-to-high	Aerobic: ≥5 days/week; 20–30 min/session Impact: ≥3 days/week; 15–20 min/session Resistance: 2–3 days/week; 30–45 min/session	/	[235]
Pelvic floor muscle training (PFMT)	PCa patients	After radical prostatectomy	High	Perform contractions and relaxation for 5–10 s in three positions: standing, sitting, and lying down, A total of 10–45 min in three groups.	12~18 weeks	[236,237]
Aerobic and resistance exercises	LC patients	During chemotherapy	Moderate-to-high	Brisk walking for 30 min per day; 5 days a week; Two resistance training sessions per week, each lasting 20 min	12 weeks	[238]
Inspiratory muscle training (IMT)	LC patients	Advanced lung cancer	Moderate	15~20 min each time; Twice a day	>4 weeks	[239]

## Abbreviations

PCa: prostate cancer; CRC: colorectal cancer; LC: lung cancer; HNC: head and neck cancer; PC: pancreatic cancer; TC: testicular cancer; HRQOL: health-related quality of life; QOL: quality of life; WHO: the World Health Organization; NCI: the National Cancer Institute; ACS: the American Cancer Society; CDC: the Centers for Disease Control and Prevention; PDA: pancreatic ductal adenocarcinoma; TCE: Traditional Chinese Exercise; TME: tumor microenvironment; MEL: melanoma; EPI: epinephrine; EC: esophageal cancer; OC: ovarian cancer; KYN: kynurenine; Fe: iron; MM: multiple myeloma; MOTS-c: Mitochondrial-derived Peptide MOTS-c; PGC-1α: Peroxisome proliferator-activated receptor-γ coactivator; ADT: androgen deprivation therapy; AMPK: AMP-activated protein kinase; mTOR: mammalian target of rapamycin; IGF-1: growth factor 1; SHBG: Sex hormone-binding globulin; IGFBP-3: Insulin-growth factor binding protein 3; KC: kidney cancer; CNS: central nervous system; DA: Dopamine; WB-EMS: Whole-body electromyostimulation; EMT: epithelial–mesenchymal transition; CTCs: Circulating tumor cells; CDCA4: Cell division cycle-associated protein 4; ROM: range of motion; NMES: neuromuscular electrical stimulation; VR: virtual reality; CRF: Cancer-related fatigue; MFSI-SF: Multidimensional Fatigue Symptom Inventory; FSS: Fatigue Severity Scale; PMR: Progressive muscle relaxation; RT: Resistance training; AT-HIIT: Moderate-intensity aerobic and high-intensity interval training; RT-HIIT: Resistance training combined with high-intensity interval training; HIIT: high-intensity interval training; HI: high-intensity exercise; LMI: low-to-moderate intensity exercise; MICT: moderate-intensity continuous training; PA: physical activity; AI:artificial intelligence; 6MWT:the 6 min walk distance values; Refs: references.
